# Molecular Background of miRNA Role in Asthma and COPD: An Updated Insight

**DOI:** 10.1155/2016/7802521

**Published:** 2016-06-08

**Authors:** Izabela Szymczak, Joanna Wieczfinska, Rafal Pawliczak

**Affiliations:** Department of Immunopathology, Faculty of Medical Science and Postgraduate Training, Medical University of Lodz, Zeligowskiego 7/9 Street, Building 2, Room 122, 90-752 Lodz, Poland

## Abstract

Inflammatory airway diseases are a significant health problems requiring new approaches to the existing therapies and addressing fundamental issues. Difficulties in developing effective therapeutic strategies might be caused by lack of understanding of their exact molecular mechanism. MicroRNAs (miRNAs) are a class of regulators that already revolutionized the view of gene expression regulation. A cumulating number of investigations show a pivotal role of miRNAs in the pathogenesis of asthma, chronic obstructive pulmonary disease (COPD), or airway remodeling through the regulation of many pathways involved in their pathogenesis. Expression changes of several miRNAs have also been found to play a role in the development and/or improvement in asthma or COPD. Still, relatively little is known about the role of miRNAs in inflammatory disorders. The microRNA profiles may differ depending on the cell type or antigen-presenting cell. Based on the newest literature, this review discusses the current knowledge concerning miRNA contribution and influence on lung inflammation and chosen inflammatory airway diseases: asthma and COPD.

## 1. Introduction

Noncoding RNAs, housekeeping RNAs, long noncoding RNAs, and small noncoding RNAs (endogenous short interfering RNA and microRNAs (miRNAs)), have emerged as important molecules in lung disease [1], out of which, in lung diseases, the miRNAs are most commonly investigated. miRNAs are short, endogenous, single-stranded, noncoding, and consisting of 18–25 nucleotides RNAs and evolutionary conserved sequences that regulate both expression of genes and gene networks [2]. They are capable of inducing degradation of messenger RNA (mRNA) and/or repression of mRNA translation. Targets for miRNAs may be approximately 60% of mRNAs [3]. miRNAs have a role in controling the signaling pathways in many types of cells and in phenotype and development of cells of the immune system as well as regulation of the inflammatory response in many tissues [4].

The biogenesis of miRNA begins in the nucleus. The primary miRNAs are transcribed by RNA polymerase II [2] and cleaved by Drosha, a nuclear RNAse III enzyme [5–7]. The resulting pre-miRNA is transported into the cytoplasm and processed into double-stranded miRNA by Dicer, a RNAse III enzyme. One strand of this duplex represents the mature miRNA and is assembled into the RNA-induced silencing complex (RISC), while the other strand of this duplex is degraded. miRNA incorporated into the RISC regulates expression of genes by translational repression or degradation of mRNA, which depends on complementarity of sequence between mRNA 3′ untranslated region and miRNA 5′ region [2, 8, 9]. This review focuses on relationship between miRNAs and inflammatory pulmonary diseases. Additionally, the association of miRNAs with molecular pathways involved in inflammation coexisting with pulmonary diseases was summarized.

## 2. Inflammation

Inflammation is a process aiming at neutralizing or removing harmful factors such as damaged cells, allergens, irritants, pathogens, toxins, and initiating healing process in the tissue. In healthy individuals, the immune system is very efficient in detecting even a very small number of pathogens as well as eliminating them, before they have reached harmful numbers. However, inadequate or excessive responses may lead to severe inflammation as well as tissue damage [10–12].

It has been demonstrated that several miRNAs play an important role in the regulation of different inflammatory processes related to asthma, COPD (chronic obstructive pulmonary disease) and airway remodeling [13–15]. The need for research on microRNA molecules is confirmed by the fact that many studies have been registered as clinical trials. Over 20 clinical trials, evaluating miRNA in inflammation, are registered, 6 of which are concerned only about airway diseases, whereas 6 and 8 trials tackle problems of asthma and obstructive lung disease, respectively (https://clinicaltrials.gov/).

## 3. Asthma

Asthma is a respiratory disease, characterized by limitation of air flow and chronic inflammation due to persistent infiltration of mast cells and eosinophils, related to poor Th2 response or not associated with this immune process [16]. Environmental and genetic factors play a role in the pathogenesis of the disease. IL-4, IL-5, IL-9, and IL-13 are Th2 cytokines involved in hypersensitivity of airway, elevated secretion of mucus, and increased IgE levels and infiltration of eosinophils [17].

Recently, the number of studies on miRNA role in asthma has elevated, indicating various expressions of miRNA in different models of asthma. Despite the fact that the methods of experimental induction of asthma are different, the same phenotypes are demonstrated by them, like Th2-related inflammation, eosinophil airway recruitment, elevated airway hypersensitivity, and secretion of mucus. Different expression of 21 miRNAs in allergic asthma and healthy individuals has been shown in mouse models of allergic asthma [18]. Some studies demonstrated that the expression of miR-21 increased in asthma models and was related to Th2 response and IL-12 expression [18, 19]. It has been observed in* Aspergillus fumigatus*, ovalbumin, house dust mite, and lung specific IL-13 models of asthma [18].

Lower levels of eosinophilia, decreased IL-4, and elevated INF-*γ* in bronchoalveolar lavage fluid were observed in ovalbumin-induced model of asthma in mice with lack of miR-21, after allergens' exposure, compared to the controls [19]. Additionally, lack of miR-21 in CD4+ T cells resulted in decreased IL-4 and increased INF-*γ* levels [19].

**Figure 1:**
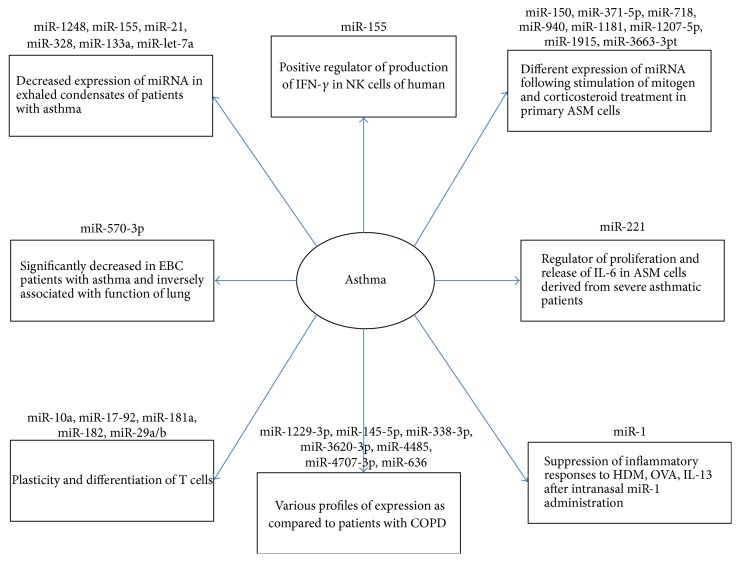
The role of miRNAs in asthma [20, 22, 24–30], in recent studies.

Various asthma models were documented to have different expression of miRNAs. Wang et al. identified different expression of miR-145-5p, miR-636, miR-338-3p, miR-4485, miR-1229-3p, miR-4707-3p, and miR-3620-3p in the serum of patients with asthma, compared to patients with COPD [20]. A few studies demonstrated different expression of 11 miRNAs in the exhaled breath condensate (EBC) from patients with asthma compared to healthy individuals [21]. Roff et al. also detected decreased levels of miR-570-3p in EBC and in the serum from the patients with asthma. Inverse correlation between levels of miR-570-3p and lung function was documented, based on the observation of diverse effect on chemokine and cytokine expression in human epithelial cells of airways with upregulation of IL-6, tumor necrosis factor (TNF), chemokine (C-C motif) ligand 4 (CCL4), CCL5 in response to TNF, suppression of TNF-induced chemokine (C-X-C motif) 8, and CCL2 [22]. Significant differences in exosomal miRNA from bronchoalveolar lavage fluid was observed in mild asymptomatic asthma patients in comparison with healthy individuals [23].  Figure 1 shows the role of miRNAs in asthma.

Expression of miR-126 and miR-21, identified in epithelial cells of airway in patients on inhaled corticosteroids, was decreased whereas it was upregulated in patients with asthma. Thus, the miRNAs may be related to asthma and may be a biomarker of the therapy [32].

Different expression of miRNAs, mRNAs, and ncRNAs was detected in human primary airway smooth muscle (ASM) cells following corticosteroid treatment and mitogen stimulation [28]. Altered miR-221 expression was documented in ASM cells from patients with nonsevere asthma and those with severe asthma and healthy individuals. miR-221 also regulates inflammatory state and aberrant proliferation observed in ASM cells [26]. The regulatory role of miR-146a in inflammation in ASM cells from patients with asthma was confirmed. Decreased RNA binding protein human antigen R (HuR) expression is related to downregulation of cyclooxygenase-2 (COX-2)* via* miR-146a [33]. Proinflammatory cytokines induced expression of miR-146a and miR-146b in ASM cells; expression of miR-146a was higher in cells derived from patients with asthma. miR-146a may be determined as a negative endogenous regulator in ASM cells. Thus, the potential candidate used in anti-inflammatory asthma treatment may be miR-146 mimics. The direct miR-570-3p target is also HuR. Comer et al. reported that miR-155 is involved in *β*2-adrenoceptor hyperresponsiveness and regulates COX-2 and secretion of prostaglandin E2 from ASM cells of humans [34]. Positive correlation of miR-155 and COX-2 expression in ASM cells from asthmatic patients was observed. miR-10a is a regulator of mitogen-induced proliferation of ASM* via* targeting the phosphoinositide 3-kinase (PI3K) pathway by inhibition of the subunits of PI2K and PIK3CA expression [35].

Exacerbation of asthma may be caused by many gaseous components of environmental pollutants, that is, ozone. Ambient particulate matter and particles of diesel exhaust may upregulate thymic stromal lymphopoietin (TSLP), which is cytokine linking Th2 adaptive immune and innate disorders, as well as miR-375 expression in human epithelial cells of bronchi [27]. Upregulated TSLP in human epithelial cells of bronchi is associated with interaction between miR-375 and regulatory effects upon the aryl hydrocarbon mRNA. Ozone is related to many adverse effects on health and elevated levels of miRNAs including miR-199a, miR-199b-p5, miR-132, miR-424, miR-223, miR-143, miR-222, miR-582-5p, miR-25, and miR-145 expression in bronchial airways of humans [36]. Environmental dust pollution may cause dyspnea, linked with alterations in expression of miRNA in bronchoalveolar lavage fluid, urine, and serum, found in military personnel serving in Afghanistan and Iraq. Interestingly, different overexpression of miR-371-5p was observed in these fluids [37].

Not only expression of miRNA but also genetic background may be related to response to the drug treatment. The miR-148a, miR-148b, and miR-152 inhibited expression of major histocompatibility complex, classes I and G, and single nucleotide polymorphism in the 3′-UTR region of HLA-G modulated binding of miRNA. Individuals with the G minor allele of the single nucleotide polymorphism showed reduced exacerbation of asthma in comparison with the individuals who did not carry the G minor allele after treatment with statins [38].

## 4. COPD

COPD and asthma are diseases that cause chronic inflammation of the airways but have distinct characteristics. COPD is characterized by the presence of chronic obstruction and is not very reversible to airflow associated with an anomalous inflammatory reaction, mainly tobacco smoke. In COPD the cellular composition of the airway inflammatory infiltrate contains mainly neutrophils, macrophages, and lymphocytes. In contrast, asthma is partially conditioned by genetic factors and runs its course with bronchial hyperresponsiveness and airflow obstruction, which either totally or partially reverses spontaneously or by medicinal action. Eosinophils are the most prominent inflammatory cells in asthma, with mast cells, lymphocytes, and macrophages playing important but less prominent roles. The contrasting inflammatory phenotypes of asthma and COPD have important implications for clinical and physiologic manifestations of disease, as well as for therapy [39, 40].

**Figure 2:**
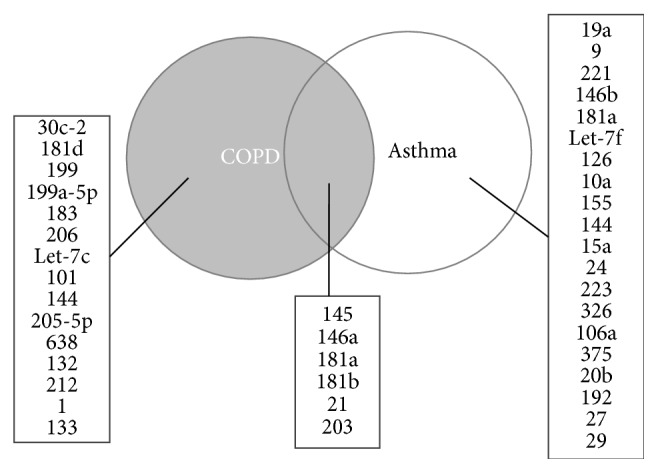
Venn diagram of microRNAs (miRNAs) associated with chronic obstructive pulmonary disease (COPD) and/or asthma. The lists show the miRNAs associated with each component of the diagram, based on Molina-Pinelo et al. [31].

Similarly to asthma, a pathophysiological role for miRNAs in COPD has been suggested in several studies, in which differential expression of miRNAs has been identified in lung cell (Figure 2) [41, 42].

Cao et al. detected increased expression of miR-183, miR-200b, and miR-200c in the peripheral blood in patients with COPD but found no significant difference between different severities of COPD. Their study suggests that increased expression of miR-183 in COPD patients' peripheral blood might be involved in regulation of BKCa*β*1 expression, which in turn might be correlated with the severity of the disease. BKCa*β*1 and miR-183 could be therefore considered promising biomarkers for clinical diagnosis and treatment of COPD [43].

Furthermore, Donaldson et al. found that, in patients with stable COPD, plasma levels of muscle-specific miRNAs are increased, suggesting that muscle wasting or turnover is increased even in samples from patients with stable COPD [44]. The study showed that plasma levels of muscle-specific miR-499 are associated with nuclear NF-*κ*B p50 in mild/moderate COPD, whereas, in severe and very severe disease, miR-206 and miR-133 are associated with circulating cytokines. This observation raises the possibility that inflammation is an important driver of wasting in this patient subgroup.

The expression of numerous inflammatory mediators associated with COPD pathogenesis is controled by NF-*κ*B. In COPD, higher levels of activated NF-*κ*B are observed in the bronchial biopsies and inflammatory cells of individuals [45]. Moreover, plasma levels of muscle-specific miR-499, miR-133, and miR-206 were elevated in COPD patients compared to the healthy controls, but, among the patients, the highest plasma levels were associated with better lung function [44]. This paradox was explained by differences in the muscle pool in patients with mild versus severe disease to release miRNA. The decrease in miR-499 observed in this study in patients with severe lung function impairment is consistent with observations from patients with lung cancer in whom low plasma levels of miR-499 were a predictor of reduced survival, perhaps as a result of cachexia [46]. As plasma miR-499 levels are directly correlated with nuclear NF-*κ*B p50 in COPD, this might suggest that loss of type I fibres or export of miR-499 is dependent on NF-*κ*B activation [44].

Aside from the classic transcription factors, muscle-specific miRNAs play a relevant role in the regulation of muscle development and repair after injury by targeting different pathways. In COPD, the inspiratory loads to which the respiratory muscle is continuously exposed may be a major player accounting for specific pattern of miRNA expression [47]. In the main inspiratory muscle (diaphragm) of patients with mild-to-moderate and severe COPD, expression of some muscle-specific miRNAs miR-1, miR-133, and miR-206 is downregulated. However, the expression of miR-486, miR-27a, miR-29b, and miR-181a does not differ between the patients and the controls [47].

Muscle-specific miRNAs were analyzed also by Puig-Vilanova et al. [47]. As the miRNAs (miR-1, miR-133, and miR-206) were downregulated, it is likely that epigenetic events act as biological adaptive mechanisms to better overcome the continuous inspiratory loads of the respiratory system in COPD. Histone deacetylase 2 level was upregulated in the respiratory muscle of COPD patients in this study. Another study presented data from lung tissues of COPD patients, displaying that the reduction in lung tissue histone deacetylase 2 expression may be related to the regulation of both p53 and hypoxia-inducible factor-1*α* (HIF-1*α*) [47, 48].

It has been reported that the expression of HIF-1*α* is reduced and p53 is increased in lungs from patients with COPD in comparison with the healthy controls. Mizuno et al. found overexpression of miR-199a-5p and miR-34a in the lung tissues from patients with COPD, compared to normal lungs. Moreover, increased expression of miR-34a was induced by upregulation of p53, and HIF-1*α* protein expression was inversely correlated with the expression of miR-199a-5p. The expression of miR-34a and miR-199a-5p was related to lung disease severity; the authors found also correlation between the expression of miR-34a and miR-199a-5p [49]. These data might suggest that miR-34a and miR-199a-5p not only contribute to COPD pathogenesis but also may affect HIF-1*α*-dependent lung structure maintenance program.

Distinct miRNA expression profiles were identified and validated in the study published by Molina-Pinelo et al. for each pathological group, involving 66 differentially expressed miRNAs [31]. miR-132 and miR-212 were upregulated in patients with COPD; however, no apparent correlation was observed between the burden of cigarette smoking and expression patterns of the miRNAs. Moreover, the authors also found a negative correlation between *α*1-antitrypsin mRNA and miR-132-212 cluster expression. Consistent with these findings, *α*1-antitrypsin mRNA has been recently described as a target for the miR-132-212 cluster, and its deficiency has been involved in COPD development [50, 51].

Diseases due to cigarette smoke exposure, including COPD, are associated with chronic inflammation typified by the increased expression of COX-2 protein. RelB is a protein that suppresses cigarette smoke induction of COX-2 through an unknown mechanism [52]. Zago et al. showed that endogenous expression of miR-146a in murine lung fibroblasts is dependent on RelB expression (which plays a role in reducing cigarette smoke-induced COX-2 protein expression). Moreover, suppression of cigarette smoke-induced COX-2 protein occurs* via* miR-146a, so possibly a functional association exists between RelB and miR-146a in the suppression of COX-2 protein by cigarette smoke exposure [52]. Cigarette smoke contributes to COPD by inciting inflammation, recruiting T cells, macrophages, and neutrophils to the lung, in part* via* the induction of inflammatory mediators, including COX-2; therefore miRNA participation in this process might be a promising issue.

COPD is a heterogeneous disease, and it is possible that some miRNAs may be differently expressed only in a subset of patients with COPD. From a clinical perspective, the targeting of noncoding RNAs as a novel therapeutic approach requires a deeper understanding of their function and mechanism of action. However, in the short term, changes in miRNA are likely to be of use as biomarkers for disease stratification and/or assessment of drug action [1, 39].

## 5. Airway Remodeling

ASM cells and bronchial epithelium are considered a key player in coordinating airway wall remodeling. Current therapeutics for asthma are effective in reducing inflammation; however, many therapies even long term pharmacological therapies do not reprogram the underlying immune deviation that drives the pathology. For this reason, there is a crucial need for developing new strategies to decrease or reverse the ongoing remodeling process [53, 54]. Airway remodeling is amplified by profibrotic mediators, such as transforming growth factor-*β* (TGF-*β*), which plays a cardinal role in various models of fibrosis [55].

Haj-Salem et al. proved that proliferation rate of epithelial cells of severe asthmatic patients is higher than in controls and mild asthmatics and that miR-19a increases proliferation in severe asthma through the downregulation of TGF-*β* receptor 2 [56]. They demonstrated that miR-19a itself is specifically increased in airway epithelial cells of severe asthmatics in comparison with mild asthmatic and healthy subjects. This is significant, as miR-19a belongs to the cluster miR-17~92 that plays a key role in the control of cell cycle and cell death. Moreover, repressed expression of miR-19a increased Smad3 phosphorylation through TGF-*β* receptor 2 signaling and abrogated bronchial epithelial cell proliferation [56].

Transforming growth factor *β*1 (TGF-*β*1) is an important mediator participating in the lung fibrosis development in patients with severe asthma [57, 58]. In vitro, it may also induce migration as well as proliferation of smooth muscle cells [59, 60]. Because of the key effects of TGF-*β*1 in these processes, it may be used as a model of remodeling and inflammation [61].

The effect of TGF-*β* is mediated by its interaction with transmembrane receptors. Three types of receptors can be distinguished: type I, TGF*β*R1, type II, TGF*β*R2, and type III, TGF*β*R3. Only autophosphorylated TGF*β*R2 may bind TGF-*β*. After binding with TGF-*β*, TGF*β*R1 is recruited and phosphorylated by TGF*β*R2. Then, TGF*β*R1 initiates transduction of signal that is mediated by Smad proteins [62]. The crucial role in the regulation of proliferation of airway smooth muscle cells and remodeling of airway is played by Smad3 proteins. Chen et al. demonstrated that the expression of miR-23b is inhibited by the miR-23b inhibitor sequence transfection and increased by the miR-23b mimics sequence transfection in ASMCs. The level of TGF*β*R2 expression is negatively regulated by miR-23b and miR-23b overexpression, suppressing remodeling of airway. Moreover, TGF-*β*1-induced proliferation of airway smooth muscle cells is controled by miR-23b* via* TGF*β*R2/p-Smad3 signals [63]. It has been reported that miR-23b prevents multiple autoimmune diseases through the regulation of inflammatory cytokine pathways, in which the molecule regulates a number of inflammatory cytokines, such as TGF- *β*1, NF-*κ*B, TNF, IL-1, and IL-17 [63].

There is an emerging evidence that miRNAs are important regulators of gene expression in the immune system, and this is supported by recent studies demonstrating a role for miRNAs in allergic airway inflammation [18, 64].

Allergen-induced inflammation is associated with remodeling responses in the airways of asthmatic patients, and the Th2 cytokines IL-4 and IL-13 play an important role in this process. Malmhäll et al. reported that miR-155 is involved in the regulation of allergen-induced Th2-mediated eosinophilic inflammation in the airways [64]. miR-155 deficiency resulted in diminished eosinophilic inflammation and mucus hypersecretion in the lungs of allergen-sensitized and allergen-challenged mice compared to WT control animals. Furthermore, the transcription factor PU.1, a negative regulator of Th2 cytokine production, was upregulated in the airways of allergen-challenged miR-155 knockout mice compared to the controls. The authors also demonstrated that miR-155 is involved in the local regulation of Th2 responses in allergen-induced eosinophilic airway inflammation and highlighted the role of miR-155 as a potential target in patients with allergic inflammatory diseases [64].

Nonreceptor tyrosine kinase and Abelson tyrosine kinase (c-Abl) play an important role in regulating actin cytoskeleton that is important in many cellular functions such as migration and adhesion of cells [65–67]. Recently, it has been known that c-Abl plays a role in regulation of smooth muscle cell proliferation, which contributes to the development of airway remodeling in chronic asthma. Expression of c-Abl is increased in asthmatic airway smooth muscle cells, and the factor regulating c-Abl expression is miR-203 [23]. Activated c-Abl induces polymerization of actin regulating activation and recruitment of Raf-1. Then, activated Raf-1 induces ERK1/2 and MEK1/2 enhancing proliferation of smooth muscle cells. However, c-Abl does not activate AKT that is also protein kinase implicating survival and growth of cells [68–71].

Based on the results of animal studies, it may be suggested that c-Abl may trigger pathogenesis of asthma [72, 73]. Expression of c-Abl is upregulated in cells of airway smooth muscle from asthmatic tissue of smooth muscle derived from animal model of asthma. c-Abl knockout suppresses allergen-induced airway remodeling in animal model of asthma [72]. Attenuation of airway thickening was also observed after treatment with inhibitor of c-Abl, imatinib in chronic animal model of asthma [74]. According to Levänen et al. miR-203 regulates smooth muscle cell proliferation by controling c-Abl expression, which in turn modulates the activation of ERK1/2, which was confirmed by the fact that exposure to the miR-203 inhibitor increased the level of c-Abl in these cells. Moreover, the levels of miR-203 were shown to be diminished in asthmatic airway smooth muscle cells in this study [23]. Liao et al. demonstrated that miR-203 downregulates c-Abl expression, and PDGF-induced HASM cell proliferation, and reduces PDGF-induced ERK1/2 phosphorylation but does not affect phosphorylation of AKT in human airway smooth muscle cells. miR-203 expression is decreased in asthmatic human airway smooth muscle cells [75].

What is interesting, miR-203 has also been found overexpressed in the lung and blood samples of smokers. Shi et al. demonstrated that miR-203 functions as an immune response inhibitor through targeting TAK1 and PIK3CA [76]. Higher miR-203 expression and weaker NF-*κ*B signaling activation were also detected in the COPD-diseased bronchial/tracheal epithelial cells, when stimulated by LPS. This particular micro-RNA deserves special attention as certainly it has a strong connection with airway diseases [76].

Recent study by Liu et al. displayed that miR-21 targeting may have detrimental effects on asthmatic airway remodeling [77]. According to the authors, miR-21 is significantly upregulated in smooth muscle cells of asthmatics, compared to nonasthmatic cells. The association of miR-2 with airway remodeling seems unquestionable, as the data indicate that miR-21 overexpression increased the proliferation and migration of ASM cells, which play a crucial role in asthma airway remodeling. Furthermore, miR-21 overexpression decreased the level of PTEN (phosphatase and tensin homolog) and activated the phosphoinositide 3-kinase (PI3K)/AKT pathway, while inhibition of miR-21 expression increased PTEN expression [77]. PTEN signaling pathway which is tightly associated with another targeted pathway, the PI3K/AKT pathway, leads to implementation of growth programs in cells from asthmatic patients. Moreover, the transcripts of six genes of this pathway belong to the list of 38 major targets for differently expressed miRNAs [78]. The phosphoinositide 3-kinase (PI3K) is protein catalyzing phosphorylation of phosphoinositides in 3-OH position as well as controling various intracellular signaling pathways* via* generated lipids. Selective inhibitors of PI3K decrease inflammation and several characteristics of disease in animal models. It has been proposed that PI3K/AKT inhibitors may be a novel therapy in the treatment of COPD and asthma. PI3K/AKT pathway is a cellular pathway involved in growth, differentiation, and survival of cells as well as expression of proteins and comprises also 3′-phosphoinositide dependent kinase-1 (*PDK-1*) [79].

The data published by the same team. Liu et el., revealed that another miRNA, miR-138, reduced proliferation of human airway smooth muscle cells. It also directly suppresses* PDK-1* (3′-phosphoinositide dependent kinase-1) expression by targeting the 3′-UTR of the gene. miR-138 controls ASM proliferation through directly inhibiting the PI3K pathway [80]. Thus,* PDK-1* gene has been identified as a direct target of miR-138 and is a key component in PI3K/AKT signaling, which is a pathway regulating ASM cell proliferation.

It has been suggested that numerous components of the PI3K pathway play crucial roles in the expression and activation of inflammatory mediators, inflammatory cell recruitment, immune cell function, airway remodeling, and corticosteroid insensitivity in chronic inflammatory respiratory diseases.

Aberrant inflammatory and remodeling responses in the lungs of asthmatic patients are thought to stem from deregulated gene expression, although the precise mechanism or mechanisms are still elusive [78]. Recent signaling pathway analysis demonstrated involvement of 38 miRNA-targeted mRNAs in increased cell proliferation through PTEN and PI3K/AKT signaling pathways [78]. Among them is the* PPARG* gene, known to be involved in airway inflammatory and remodeling responses, as well as adenosine A2b receptor (ADORA2B). Adenosine is a powerful bronchoconstrictor of asthmatic airways and not only acts as an inflammatory mediator in asthmatic patients but also participates in airway wall remodeling.

Relatively very few miRNAs have been studied in detail and, hence, the precise biological relevance remains yet to be determined. miRNAs have also been implicated to play a major role in airway disease regulation.

## 6. miR-27b and 1,25(OH)_2_D_3_

Pulmonary fibroblasts play a crucial role in maintenance and formation of structure as well as function of lung and remodeling and repair of tissue that are crucial characteristics of chronic lung diseases, including pulmonary fibrosis, COPD, and asthma [81–86]. TGF-*β*1 is produced by many types of cells and suggested to influence cell function, growth regulation, proliferation, apoptosis, differentiation, cell movement, and secretion and deposition of extracellular matrix. Moreover, TGF-*β*1 regulates function and phenotype of fibroblasts [87–90].

Active metabolite of vitamin D, 1,25(OH)_2_D_3_, is able to suppress tissue remodeling response mediated by TGF-*β* in fibroblast of lung and block TGF-*β*1-mediated myofibroblastic transformation of fibroblasts. Some studies demonstrated association of TGF-*β* and vitamin D signaling pathway. Vitamin D receptor (VDR) binds with Smad3 (MH1 domain) strengthening transactivation of Smad3 induced by ligand. Thus, active metabolite of vitamin D influences TGF-*β* signaling pathway* via* alteration of Smad3 and VDR levels. VDR may be determined as a negative regulator of TGF-*β*-induced fibroblast differentiation. Expression of TGF-*β*1-induced miR-27b is downregulated by 1,25(OH)_2_D_3_ in human fibroblast of lung. VDR expression in human fibroblast of lung is regulated* via* miR-27b that has capabilities of targeting VDR 3′-UTR and downregulation of VDR protein expression contributing to human lung fibroblast differentiation. In summary, active metabolite of vitamin D suppresses TGF-*β*1-induced differentiation of human lung fibroblasts by miR-27b targets 3′-UTR of VDR; thus it may be a potential novel strategy of treatment in pathways of differentiation [91].

## 7. Conclusion

Animal models of asthma and isolated airway cell studies showed the role of miRNA in inflammation of airway and related asthma and COPD as well as remodeling and inflammation-related signaling pathways. Evidence presented in this review supports the idea that miRNAs are emerging as novelty therapeutic and diagnostic targets in inflammatory lung diseases.
